# The use of self-reported symptoms as a proxy for acute organophosphate poisoning after exposure to chlorpyrifos 50% plus cypermethrin 5% among Nepali farmers: a randomized, double-blind, placebo-controlled, crossover study

**DOI:** 10.1186/s12940-016-0205-1

**Published:** 2016-12-13

**Authors:** Dea Haagensen Kofod, Erik Jørs, Anshu Varma, Shankuk Bhatta, Jane Frølund Thomsen

**Affiliations:** 1 Department of Occupational and Environmental Medicine, Bispebjerg Frederiksberg Hospital, University of Copenhagen, Bispebjerg Bakke 23, Entrance 20F, 2400 Copenhagen NV, Denmark; 2 Department of Occupational and Environmental Medicine, Odense Hospital, University of Southern Denmark, Odense, Denmark; 3 International Centre for Occupational, Environmental and Public Health (ICOEPH), Odense Hospital, University of Southern Denmark, Odense, Denmark; 4 Dialogos, Noerrebrogade 52C, Copenhagen N, Denmark; 5 Institute of Medicine, Tribhuvan University of Nepal, Kathmandu, Nepal

**Keywords:** Farmers, Organophosphate, Acute pesticide poisoning, Symptoms, Plasma cholinesterase, Nepal

## Abstract

**Background:**

Previous studies stating a high prevalence of occupational acute pesticide poisoning in developing countries have mainly relied on measurements of the rather non-specific self-reported acute pesticide poisoning symptoms. Only a few studies have measured the biomarker plasma cholinesterase (PchE) activity, in addition to the symptoms, when assessing occupational acute pesticide poisoning. This study evaluated self-reported symptoms as a proxy for acute organophosphate poisoning among Nepali farmers by examining self-reported acute organophosphate poisoning symptoms and PchE activity in response to occupational acute organophosphate exposure.

**Methods:**

We performed a randomized, double-blind, placebo-controlled, crossover trial among 42 Nepali commercial vegetable farmers. The farmers were randomly assigned (ratio 1:1) to a 2-h organophosphate (chlorpyrifos 50% plus cypermethrin 5%: moderately hazardous) spray session or a 2-h placebo spray session, and after 7 days’ washout, the farmers were assigned to the other spray session. Before and after each spray session farmers were interviewed about acute organophosphate poisoning symptoms and PchE activity was measured. Analyses were conducted with a Two Sample T-test and Mann Whitney U-test.

**Results:**

We found no difference in the symptom sum or PchE activity from baseline to follow up among farmers spraying with organophosphate (symptom sum difference −1, *p* = 0.737; PchE mean difference 0.02 U/mL, *p* = 0.220), placebo (symptom sum difference 9, *p* = 0.394; PchE mean difference 0.02 U/mL, *p* = 0.133), or when comparing organophosphate to placebo (symptom *p* = 0.378; PchE *p* = 0.775). However, a high percentage of the farmers reported having one or more symptoms both at baseline and at follow up in the organophosphate spray session (baseline 47.6%, follow up 45.2%) and placebo spray session (baseline 35.7%, follow up 50.0%), and 14.3% of the farmers reported three or more symptoms after the organophosphate spray session as well as after the placebo spray session.

**Conclusion:**

We found a general presence of acute organophosphate symptoms among the farmers regardless of organophosphate exposure or poisoning. Thus, self-reported acute organophosphate symptoms seem to be a poor proxy for acute organophosphate poisoning as the occurrence of these symptoms is not necessarily associated with acute organophosphate poisoning.

**Trial registration:**

ClinicalTrials.gov, NCT02838303. Registered 19 July 2016. Retrospectively registered.

**Electronic supplementary material:**

The online version of this article (doi:10.1186/s12940-016-0205-1) contains supplementary material, which is available to authorized users.

## Background

Exposure to pesticides is considered as one of the most important occupational health risks among farmers in developing countries [1, 2]. An estimated 25 million farmers suffer from acute pesticide poisoning annually in developing countries, making pesticide poisoning a major global health problem [2–4]. Some of the most widely used pesticides of farmers worldwide, like organophosphates, cause acute pesticide poisoning by inhibiting cholinesterase enzymes. This results in excessive stimulation of nicotinic and muscarinic receptors causing the symptoms of acute pesticide poisoning like headache, dizziness, weakness, vomiting, diarrhea, bradycardia, dyspnea, ataxia, paralysis and worst case death [5, 6].

Previous studies stating a high prevalence of occupational acute pesticide poisoning among farmers in developing countries [7–13] have mainly relied on measuring self-reported acute pesticide poisoning symptoms when assessing the prevalence. However, acute pesticide poisoning symptoms are rather non-specific and can have multiple causes such as heat exhaustion, dehydration, or various diseases, and there may be some degree of recall and report bias when relying on self-reported symptoms solely. Thus, measuring cholinesterase enzyme activities, in addition to acute pesticide poisoning symptoms, is suggested when assessing occupational acute pesticide poisoning. Plasma cholinesterase (PchE) activity is considered a valid biomarker of acute pesticide exposure and poisoning [5, 6, 14], however, to date only a few studies have measured PchE activity in relation to occupational pesticide poisoning [15–19] and these studies have mainly examined occupational chronic pesticide poisoning.

The purpose of this study was to evaluate self-reported symptoms as a proxy for acute organophosphate poisoning by examining self-reported acute organophosphate poisoning symptoms and PchE activity in response to occupational acute organophosphate exposure among farmers in Nepal.

## Methods

### Study design

We performed a randomized, double-blind, placebo-controlled, crossover trial collecting baseline and follow up measures of self-reported acute organophosphate poisoning symptoms and PchE activity in Nepali farmers randomized to either Group A (farmers spray with organophosphate in their first spray session and placebo in their crossover spray session) or Group B (farmers spray with placebo in first their spray session and organophosphate in their crossover spray session). The data presented was gathered by the principal investigator and a local partner over a 2-month period from September to November 2014.

### Study area, population and recruitment

Very few studies examining occupational acute pesticide poisoning have been carried out in Nepal, however, the increasing pesticide consumption and health hazardous pesticide behavior among Nepali farmers, suggest a risk of significant exposure and poisoning [9, 20, 21]. This study was conducted in two purposely selected villages, Sukranagar and Jagatpur, in the Chitwan District of Nepal, where vegetable production is intensive and pesticide use extensive [22]. The study participants consisted of commercial vegetable farmers. Inclusion criteria were: male, minimum age 18 years, hand pressured backpack sprayer usage, and used to spray with moderately to extremely hazardous pesticides according to the World Health Organization’s (WHO) Pesticide Hazard Classification [23]. Exclusion criteria were: usual personal protective equipment use (respirator/mask with particulate filter, face shield, googles, gloves, boots, plastic poncho), unwilling to stay pesticide-free 7 days prior to each of the two spray sessions, or medical conditions interfering with PchE activity (liver disease, acute infection, chronic malnutrition, heart attack, cancer, obstructive jaundice, inflammation caused by various diseases, or use of pyridostigmine drugs) [24, 25].

The farmers were contacted through the management in their respective Vegetable Farmer Cooperative, who invited the farmers to a meeting regarding the study. The farmers were informed about the purpose of the study, its advantages and disadvantages, and were asked to volunteer for the study. All participating farmers signed an informed consent prior to participation. The study was approved by Nepal Health Research Council’s Ethical Review Board, Reg. no. 162/2014.

### Intervention

Each farmer participated in two spray sessions, which each had an average duration of 2 h (minimum 1 h and 45 min; maximum 2 h and 15 min). Each spray session was preceded by minimum 7 days’ washout in which the farmers had to stay pesticide-free. In the organophosphate spray session the farmers sprayed with chlorpyrifos (organophosphate) 50% plus cypermethrin (pyrethroid) 5% EC, WHO Class II: Moderately hazardous, in the farmers’ usual dilution ratio. In the placebo spray session the farmers sprayed with the biopesticide Multineem, WHO Class U: Unlikely to present acute hazard in normal use. The dilution ratio for Multineem was 2 mL per L water. Multineem was purposely selected, as it is similar to the organophosphate pesticide in appearance and odor.

### Data collection

A structured questionnaire interview and blood tests were conducted in each farmer’s home just before (baseline) and about 30 min after (follow up) each spray session by the local partner. Observations were made based on a checklist during each farmer’s spray session by the principal investigator and the local partner. The structured questionnaire interview and observation checklist were developed based on studies applying similar methods [13, 21, 26, 27]. Under the supervision of the principal investigator, the structured questionnaire interview was conducted face-to-face in Nepali by the local partner, and back translated into English later on the same day.

Self-reported acute organophosphate poisoning symptoms were assessed with the baseline question “Have you suffered from any of the following symptoms in the last 7 days?” and follow up question “Have you suffered from any of the following symptoms during or after spraying today?”. The definition of symptoms was based on WHO’s standardized list of clinical presentations of acute organophosphate poisoning [2]. Some of these clinical terms were considered difficult for the farmers to understand, therefore, these terms were translated into more understandable terms, the final list of self-reported acute organophosphate poisoning symptoms being: headache, dizziness, skin irritation, extreme tiredness, weakness, anxiety, excessive sweating, trembling hands, vomiting, diarrhea, abdominal pain, blurred vision, paralysis, salivation, tearing, lack of coordination, respiratory difficulties, confusion.

PchE activity was measured with a blood test using a Test-mate Che Cholinesterase System (Model 400) with a PchE Plasma Cholinesterase Assay Kit (Model 470). This system is considered to involve a reliable test method for measuring PchE inhibition of farmers due to organophosphate exposure [28, 29]. The blood test was performed in accordance with the Instruction Manual [30]. In short, after cleaning the farmer’s fingertip with water, soap and an alcohol swap, the fingertip was punctured with a sterile blood lancet, and a 10 microliter capillary tube was filled with blood. The capillary tube was inserted into an assay tube, and afterwards PchE Plasma Cholinesterase Reagent was added. A photometric analyzer calculated the test results. Given the large inter- and intraindividual variation of PchE, it is necessary to establish individual baseline PchE activities, adopted as a reference. The interindividual coefficient of variation is about 15–25%, whereas the intraindividual coefficient of variation is about 6–9% [31, 32]. PchE activities after exposure should be expressed as percentage change with respect to the individual baseline [31]. A 50% PchE inhibition from baseline is suggested as the level of acute overexposure and poisoning [5].

Descriptive variables partly included interviews about the background characteristics age, height, weight, marital status, educational level, farming experience, pesticide experience, medical condition, medication, tobacco status, alcohol consumption, names of pesticides the farmer usually uses, and partly observations of temperature, clothing during spraying, work practices during and after spraying, total area of spraying and if the backpack leaked. In addition to the PchE activity, we measured a hemoglobin (Hb) level with the Test-mate Che Cholinesterase System to evaluate whether the spray session resulted in dehydration of the farmers, which would be reflected as an increase in Hb level after spraying.

The informed consent, intervention, placebo and data collection procedure were pretested on one commercial vegetable farmer from Sukranagar. During pretesting of the intervention and placebo both the farmer and the local partner were unaware of the pesticide assignment (placebo) chosen by the principal investigator, however, both the farmer and the local partner believed that the placebo was organophosphate.

### Sample size calculation

The sample size calculation was based on data from a previous study of 25 farmers in Chitwan whose acute organophosphate poisoning symptoms significantly increased after an organophosphate spray session (Neupane D. Pesticide exposure and its health effects among commercial vegetable farmers in Nepal. Unpublished masters’ thesis. University of Southern Denmark; 2012). The sample size was calculated using SAS 9.3 Proc Power for paired means (alpha 0.05, power 0.90). A sample size of 34 pairs was required. Forty-two farmers were enrolled taking into account possible dropouts and technical problems.

### Randomization and blinding

The participating farmers were assigned a study ID number from 1 to 42. Forty-two opaque envelopes containing a study ID card with a study ID number were prepared by the principal investigator. A person not involved in the project (Khilendra R. Chaudhary) randomly allocated the envelopes to two buckets assigned Group A or Group B (ratio 1:1). The person wrote the assigned group on the study ID card, put the card back into the envelope, wrote the study ID number on the envelope, sealed the envelope and placed the envelopes in numerical order. The envelopes were placed in a secure location to which only the principal investigator had access.

On the day of each farmer’s first spray session, the principal investigator brought the numerically relevant sealed envelope, and opened it prior to group assignment. The principal investigator diluted the assigned pesticide (organophosphate or placebo) into the farmer’s backpack sprayer, while the farmer and the local partner stayed at a location where they could not see the mixing site. Afterwards, the backpack was handed to the farmer closed and ready to use. This procedure was repeated every time the farmer needed a backpack refill. Thus, the principal investigator assigned all farmers to their intervention, ensuring that the local partner and all farmers remained unaware of the pesticide assignment.

### Data analysis

Double data entry, cleaning and statistical analyses were performed in SPSS version 22. *P* values lower than 0.05 were considered statistically significant. Mean (SD) was applied for parametric tests and median (IQR) for non-parametric tests. Body Mass Index was calculated as weight (in kilograms) over height squared (in meters).

To compare the farmers’ characteristics between Group A and Group B a two sample t-test was applied. If the normality assumption was violated the Mann Whitney U-test was applied. For categorical data the Pearson Chi-Square test was applied, or when more than 20% of the cells had expected values less than five, Fishers’ exact test (2×2 tables) and Likelihood Ratio (tables > 2×2) were applied.

To compare outcome measures between the organophosphate spray session and the placebo spray session, we used the model recommended by Altman and colleagues for analyzing crossover trials consisting of two sample t-tests or Mann Whitney U-tests [33]. First, we applied the tests to assess whether there was any period effect or treatment-period interaction, and given the lack of such effects, we applied the same tests to compare the two spray sessions.

To compare baseline levels within Group A and Group B respectively, and to compare baseline levels and follow up levels within the organophosphate and placebo spray session respectively, we applied the paired sample t-test when data were normally distributed and the Wilcoxon signed rank test when data were not normally distributed.

## Results

**Fig. 1:**
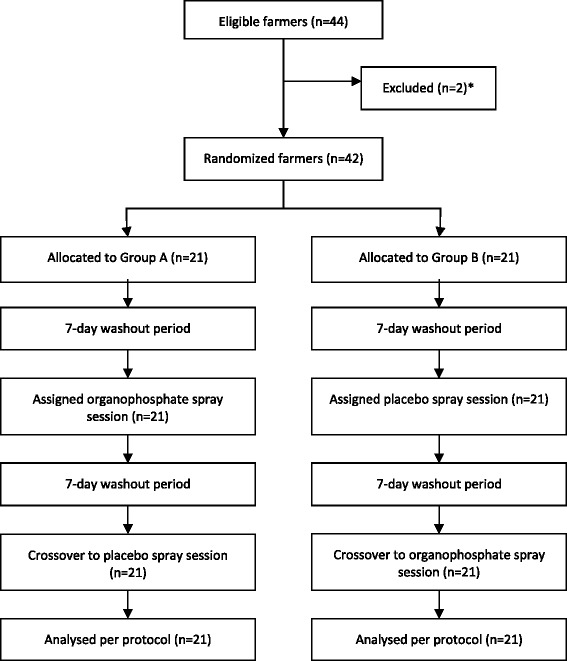
*1 excluded due to infectious leg ulcer, 1 excluded due to unwillingness to stay pesticide-free 7 days prior to each spray session

Figure 1 shows the trial profile. Of 44 eligible farmers, two were excluded due to infectious leg ulcer and unwillingness to stay pesticide-free 7 days prior to each spray session. Forty-two farmers were randomized: 21 to Group A and 21 to Group B. All randomized farmers completed their assigned initial intervention and crossover intervention.

The farmers’ characteristics are described in Table 1, which shows no statistically significant difference between Group A and Group B. Ten farmers reported having medical conditions: two reported mental problems (one medicated with fluoxetine 20 mg od. and olanzapine 5 mg od., and one medicated with fluoxetine 40 mg od.); one had high blood pressure (medicated with amlodipine 5 mg od.); seven had unmedicated, unspecific symptoms such as gastritis and difficulties in breathing. None of the conditions or medications are known to interfere with PchE activity.

**Table 1:** Characteristics of farmers

Variable	Total (*n* = 42)	Group A (*n* = 21)	Group B (*n* = 21)	*P* value*
Men, n *(%)*	42 (100)	21 (100)	21 (100)	
Age (years)				0.134^a^
Mean (SD)	38.1 (9.4)	35.9 (8.6)	40.2 (10.0)	
Education, *n (%)*				0.917^+,b^
No education	4 (9.5)	2 (9.5)	2 (9.5)	
1^st^–4^th^ grade	6 (14.3)	2 (9.5)	4 (19.0)	
5^th^–8^th^ grade	9 (21.4)	5 (23.8)	4 (19.0)	
9^th^–12^th^ grade	23 (54.8)	12 (57.1)	11 (52.4)	
Median (IQR)	10 (4.8–10)	10 (6.0–10.0)	10 (3.0–10.0)	
Involvement in farming (years), *n (%)*				0.208^+,b^
1–10	18 (42.9)	12 (57.1)	6 (28.6)	
11–20	13 (31.0)	4 (19.0)	9 (42.9)	
21–30	5 (11.9)	3 (14.3)	2 (9.5)	
> 30	6 (14.3)	2 (9.5)	4 (19.0)	
Median (IQR)	12.0 (8.0–22.3)	10.0 (6.0–21.5)	13.0 (10.0-24-0)	
Pesticide use (years), *n (%)*				0.398^+,b^
1–10	24 (57.1)	15 (71.4)	9 (42.9)	
11–20	13 (31.0)	3 (14.3)	10 (47.6)	
21–30	5 (11.9)	3 (14.3)	2 (9.5)	
Median (IQR)	10.0 (4.8–13.3)	8.0 (4.0–14.0)	11.0 (5.0–13.5)	
Marital status, *n (%)*				0.148^c^
Currently married	36 (85.7)	18 (85.7)	18 (85.7)	
Divorced/Separated	2 (4.8)	0	2 (9.5)	
Never married	4 (9.5)	3 (14.3)	1 (4.8)	
Body Mass Index (kg/m^2^)				0.505^b^
Median (IQR)	22.3 (19.8–24.9)	21.4 (18.3–25.9)	22.7 (21.4–23.4)	
Alcohol drinking, *n (%)*				0.317^d^
Yes	13 (31.0)	5 (23.8)	8 (38.1)	
No	29 (69.0)	16 (76.2)	13 (61.9)	
Smoking, *n (%)*				1.000^e^
Yes	7 (16.7)	3 (14.3)	4 (19.0)	
No	35 (83.3)	18 (85.7)	17 (81.0)	
Chewing tobacco, *n (%)*				0.212^d^
Yes	18 (42.9)	11 (52.4)	7 (33.3)	
No	24 (57.1)	10 (47.6)	14 (66.7)	

* = For difference between Group A and Group B

^+^ = Tested as a continuous variable

^a^ = Two sample t-test

^b^ = Mann Whitney U-test

^c^ = Likelihood Ratio

^d^ = Fishers’ exact test

^e^ = Pearson Chi-Square test

Table 2 describes ten of the most common pesticides usually used by the farmers when spraying their crops. A median of three different types of pesticides per farmer was reported (IQR 2.0–4.0; minimum 1; maximum 6). The most commonly used pesticide by the farmers, chlorpyrifos 50% plus cypermethrin 5% EC, was the pesticide of choice for our organophosphate spray session.

**Table 2:** Ten most common pesticides usually used by the farmers when spraying their crops (*n* = 42)

Active ingredient	Chemical type	Main use	WHO Class^a^	Reported use, n (%)
Chlorpyrifos 50% + cypermethrin 5% EC	Organophosphate and pyrethroid	Insecticide	II	38 (90.5)
Emamectin Benzoate 5% SG	Macrocyclic lactone	Insecticide	III	15 (35.7)
Carbendazim 12% + mancozeb 63% WP	Benzimidazol and dithiocarbamate	Fungicide	U	12 (28.6)
Thiamethoxam 25% WG	Neonicotinoid	Insecticide	III	8 (19.0)
Cypermethrin 10% EC	Pyrethroid	Insecticide	II	7 (16.7)
Imidacloprid 17.8% SL	Neonicotinoid	Insecticide	II	7 (16.7)
Dichlorvos 76% EC	Organophosphate	Insecticide	Ib	6 (14.3)
Triazofos 35% + deltamethrin 1% EC	Organophosphate and pyrethroid	Insecticide	II	5 (11.9)
Acetamiprid 20% SP	Neonicotinoid	Insecticide	III	5 (11.9)
Cymoxanil 8% + mancozeb 64% WP	Cyanoacetamide oxime and dithiocarbamate	Fungicide	III	5 (11.9)

^a^World Health Organization (WHO) Pesticide Hazard Classification of pesticide active ingredient: Ib Highly hazardous; II Moderately hazardous; III Slightly hazardous; U Unlikely to present acute hazard in normal use

Table 3 shows no considerable differences when comparing observations in the organophosphate spray sessions with observations in the placebo spray sessions. The median spraying time was 1.92 h (IQR 1.83–2.00 h) with organophosphate and 2.00 h (IQR 2.00–2.00 h) with placebo. In the organophosphate spray session the farmers applied a median of 103 mL (IQR 80–136 mL) chlorpyrifos 50% plus cypermethrin 5% EC per spray session (median concentration: 1.8 mL/L water (IQR 1.6–1.9 mL/L)). There was no statistically significant change in the Hb level from baseline to follow up among farmers spraying with organophosphate (mean difference −0.13 g/dL, −1.1%, *p* = 0.120) or with placebo (mean difference 0.06 g/dL, 0.5%, *p* = 0.526).

**Table 3:** Observed variables and change in Hb level in the organophosphate spray session (OP) and in the placebo spray session (PL)

Variable	OP (*n* = 42)	PL (*n* = 42)
Temperature (°C), *mean (SD)*	29.1 (3.3)	28.9 (3.5)
Area of land sprayed (Katha), *median (IQR)*	5.0 (4.0–6.6)	5.0 (3.9–6.0)
Leaking sprayer, *n (%)*	9 (21.4)	12 (28.6)
Clothing worn during spraying, *n (%)*		
Long-sleeved shirt	18 (42.9)	17 (40.5)
Long pants	20 (47.6)	20 (47.6)
Hat	21 (50.0)	23 (54.8)
Cloth mask	8 (19.0)	12 (28.6)
Work practices during and after spraying, *n (%)*		
Splash/spill on e.g. hands/feet	25 (59.5)	24 (57.1)
Spray against the wind	27 (64.3)	25 (59.5)
Walk in just sprayed crop	42 (100)	41 (97.6)
Keep nozzle close to the body	40 (95.2)	40 (95.2)
Nozzle direction in front of farmer	42 (100)	42 (100)
Adjust/repair equipment with bare hands	17 (40.5)	17 (40.5)
Eat/drink/smoke without prior hand wash	13 (31.0)	10 (23.8)
Suck/blow nozzle with mouth	5 (11.9)	2 (4.8)
Do not wash hands immediately after spraying	4 (9.5)	5 (11.9)
Do not bath/change clothes soon after spraying	14 (33.3)	14 (33.3)
Change in Hb level (g/dL) from baseline to follow up, *mean (SD)*	−0.13 (0.54)	0.06 (0.65)

Table 4 shows that there was no statistically significant change in PchE activity from baseline to follow up among farmers spraying with organophosphate (mean difference 0.02 U/mL, 1.25%, *p* = 0.220) or with placebo (mean difference 0.02 U/mL, 1.25%, *p* = 0.133), or when comparing organophosphate with placebo (*p* = 0.775). The percentage changes in PchE activity from baseline to follow up ranged between a maximum activation of 16.1% to a maximum inhibition of 10.9% after spraying with organophosphate, comparable to the changes after spraying with placebo, which ranged between a maximum activation of 17.7% to a maximum inhibition of 9.1% (data not shown, see Additional file 1).

**Table 4:** Results of the outcome measures for the organophosphate spray session (OP) and the placebo spray session (PL)

Variable	OP (*n* = 42)		PL (*n* = 42)		*P* value*
	Baseline	Follow up	Baseline	Follow up	
*PchE activity (U/mL)*					
Mean (SD)	1.58 (0.41)	1.60 (0.40)	1.58 (0.42)	1.60 (0.41)	0.775^a^
*Symptoms*					
Sum of all symptoms	42	41	31	40	0.378^b^
Number of symptoms per farmer, *n (%)*					
0	22 (52.4)	23 (54.8)	27 (64.3)	21 (50.0)	
1	11 (26.2)	10 (23.8)	6 (14.3)	10 (23.8)	
2	5 (11.9)	3 (7.1)	6 (14.3)	5 (11.9)	
≥ 3	4 (9.5)	6 (14.3)	3 (7.1)	6 (14.3)	
Distribution, *n (%)*					
Headache	6 (14.3)	10 (23.8)	4 (9.5)	10 (23.8)	
Dizziness	1 (2.4)	1 (2.4)	2 (4.8)	6 (14.3)	
Skin irritation	7 (16.7)	9 (21.4)	3 (7.1)	10 (23.8)	
Extreme tiredness	4 (9.5)	5 (11.9)	6 (14.3)	5 (11.9)	
Weakness	6 (14.3)	3 (7.1)	4 (9.5)	2 (4.8)	
Anxiety	3 (7.1)	1 (2.4)	0 (0.0)	1 (2.4)	
Excessive sweating	5 (11.9)	4 (9.5)	1 (2.4)	0 (0.0)	
Trembling hands	1 (2.4)	1 (2.4)	1 (2.4)	1 (2.4)	
Vomiting	0 (0.0)	0 (0.0)	1 (2.4)	0 (0.0)	
Diarrhea	0 (0.0)	0 (0.0)	0 (0.0)	0 (0.0)	
Abdominal pain	1 (2.4)	2 (4.8)	1 (2.4)	1 (2.4)	
Blurred vision	4 (9.5)	1 (2.4)	3 (7.1)	4 (9.5)	
Paralysis	0 (0.0)	0 (0.0)	0 (0.0)	0 (0.0)	
Salivation	1 (2.4)	1 (2.4)	1 (2.4)	0 (0.0)	
Tearing	0 (0.0)	0 (0.0)	2 (4.8)	0 (0.0)	
Lack of coordination	0 (0.0)	1 (2.4)	0 (0.0)	0 (0.0)	
Respiratory difficulties	3 (7.1)	2 (4.8)	2 (4.8)	0 (0.0)	
Confusion	0 (0.0)	0 (0.0)	0 (0.0)	0 (0.0)	

* = For difference between the organophosphate spray session and the placebo spray session

^a^ = Two sample t-test

^b^ = Mann Whitney U-test

As for PchE, no statistically significant difference was observed in the symptom sum from baseline to follow up among farmers spraying organophosphate (sum difference −1, *p* = 0.737), placebo (sum difference 9, *p* = 0.394), or when comparing organophosphate with placebo (*p* = 0.378). However, a high percentage of the farmers reported having one or more symptoms consistent with acute organophosphate poisoning both at baseline and at follow up in the organophosphate spray session (baseline 47.6%, follow up 45.2%) as well as in the placebo spray session (baseline 35.7%, follow up 50.0%). After spraying with organophosphate, 23.8% farmers increased their symptom sum, compared to 35.7% farmers after spraying with placebo (data not shown, see Additional file 1).

For both outcome measures, there was no statistically significant period effect (*p* = 0.946 for PchE, *p* = 0.171 for symptoms) or treatment-period interaction (*p* = 0.108 for PchE, *p* = 0.334 for symptoms) between the two spray sessions. Furthermore, there was no statistically significant difference in baseline levels between the organophosphate and placebo spray session in Group A (*p* = 0.350 for PchE, *p* = 0.903 for symptoms) or Group B (*p* = 0.351 for PchE, *p* = 0.320 for symptoms).

## Discussion

To our knowledge, this is the first placebo-controlled study examining self-reported symptoms as a proxy for acute organophosphate poisoning. The organophosphate spray session did not increase self-reported symptoms or decrease PchE activity, suggesting that a single 2-h spray session with a moderately hazardous organophosphate does not result in acute organophosphate poisoning among farmers in our study.

Considering that 50% PchE inhibition is suggested as the level of acute overexposure and poisoning [5], our results on PchE inhibition did not indicate that any farmer suffered from acute organophosphate poisoning. To our knowledge, only one other study [34] has assessed acute organophosphate poisoning using the same method as we did by measuring follow up right after the spray session. This study measured acetyl cholinesterase (AchE) before and after a 2-h spray session with highly to extremely hazardous organophosphates (WHO Class Ia and Ib) and in contrary to our results found a significant inhibition of AchE, suggesting that a 2-h spray session with WHO Class Ia and Ib organophosphates does produce acute health effects. The difference could be due to the difference in WHO Hazard Class, and suggests that, as an initial approach to reduce acute organophosphate poisoning, it may be of importance to at least phase out farmers’ use of WHO Class I organophosphates.

We found no considerable increase in self-reported symptoms after the organophosphate or placebo spray session, however, a high percentage of the farmers reported having one or more symptoms both at baseline and at follow up for the organophosphate spray session (baseline 47.6%, follow up 45.2%) as well as the placebo spray session (baseline 35.7%, follow up 50.0%). According to WHO’s case definition matrix of acute organophosphate poisoning [2], a person is considered a probable case of acute organophosphate poisoning if the person has a known exposure of organophosphate, a temporal cause-effect relationship, and reports three or more symptoms of acute organophosphate poisoning. Applying this matrix, we found that 14.3% of the farmers were classified as a probable case of acute organophosphate poisoning at follow up after the organophosphate spray session. However, 14.3% of the farmers also reported three or more symptoms after the placebo spray session. Furthermore, 9.5% (for organophosphate) and 7.1% (for placebo) of the farmers already reported having three or more symptoms at baseline. No farmer’s change in PchE activity indicated acute organophosphate poisoning. Thus, using self-reported symptoms to assess acute organophosphate poisoning may lead to an overestimation, as there is a general presence of symptoms consistent with acute organophosphate poisoning among the farmers regardless of organophosphate exposure. In other words, the occurrence of these symptoms is not necessarily associated with acute organophosphate poisoning, as they are non-specific, making them a poor proxy for acute organophosphate poisoning. This suggests that future studies should not solely rely on self-reported symptoms when assessing acute organophosphate poisoning but include biomonitoring to achieve sound estimates. Such a mixed approach might be helpful in identifying the most important areas of intervention to reduce acute organophosphate poisoning among farmers in developing countries.

Even though our study did not find that the farmers suffer from occupational acute organophosphate poisoning, their work practices still raise potential acute and chronic poisoning concerns. Our study, as well as other studies from developing countries [7–9, 21, 26, 35], show that farmers have unsafe work practices exposing them to pesticides. About 58% of the farmers splashed/spilled pesticides and 41% adjusted their pesticide spraying equipment with bare hands. Thus, if the farmers spray with WHO Class I pesticides, or spray for longer than the 2 h that are considered to reflect farmers’ usual spraying time [27, 34, 36], while applying unsafe work practices, a considerable risk of acute organophosphate poisoning can be expected. Furthermore, long-termed exposure to low doses of hazardous pesticides are linked to adverse health effects in immune, hematological, nervous, endocrine and reproductive systems as well as DNA damage [2, 16]. Thus, interventions promoting safe work practices reducing farmers’ hazardous pesticide exposure are needed to reduce farmers’ risk of acute pesticide poisoning and chronic sequelae of pesticide exposure.

There are several strengths of this study. Firstly, the randomized, double-blind, placebo-controlled study design makes it possible to evaluate self-reported symptoms as a proxy for acute organophosphate poisoning. Secondly, by measuring follow up just after the spray session, this study has strictly assessed acute organophosphate poisoning. Thirdly, work practices and clothing were directly observed during spray sessions thus limiting recall and report bias to the self-reported symptoms.

Major limitations are first of all the non-exposure washout period of 7 days which may have been inadequate in order for PchE to reach its true unexposed baseline activity as a study suggests this may take around 50 days [14]. However, there was no difference in the baseline levels between the organophosphate and placebo spray session for each group, indicating that the washout period was successful. To evaluate whether our farmers to some extent have been exposed with a chronic depression in PchE activity at the baseline measurements it would have been of interest to compare our baseline PchE measurements with measurements on non-exposed individuals. However, due to the wide inter-individual variation in PchE activity, we would have needed a substantial number of non-exposed individuals to establish a normal value but this was not possible within our research frame. At the same time, a reference value for PchE activity in a population comparable to our population could not be found in the literature [6, 14]. Ideally, we should have measured the farmers’ PchE levels in off-season and compared with levels within season to see if a seasonal PchE depression had taking place as previous studies from Nepal and Pakistan have found a depression in AchE and PchE activity when comparing farmers’ levels in spray-season with their levels in off-season [19, 20]. This was not possible within our study but it implies that we, more strictly speaking, may have evaluated “acute-on-chronic” pesticide exposure and poisoning. Other limitations include not involving the farmers in the pesticide mixing and loading which might have resulted in the exposure of a lower pesticide concentration than during their usual work. Lastly, the sampling technique on what may seem like a small sample size might hinder generalizations to farmers in other parts of Nepal. However, the farmers seemed representative in terms of pesticides usually used, work practices and clothing compared to findings from previous studies in Nepal [9, 20, 21], and we expect that our results will be relevant to other farmers spraying with moderately hazardous pesticides.

## Conclusions

In conclusion, self-reported symptoms seem to be a poor proxy for acute organophosphate poisoning, as there was a general presence of acute organophosphate poisoning symptoms among the farmers regardless of organophosphate exposure or poisoning indicating that the occurrence of these symptoms is not necessarily associated with acute organophosphate poisoning. Future studies are needed to determine the reproducibility and generalizability of our data.

## Additional file

Additional file 1: Number of symptoms and PchE activity at baseline and follow up, and change in symptom sum and percentage change in PchE activity from baseline to follow up for each farmer for the organophosphate spray session (OP) and the placebo spray session (PL). Description: data on symptoms and PchE activity on which conclusions of the manuscript rely. (DOCX 114 kb)

## Abbreviations

AchE: Acetyl Cholinesterase
Hb: Hemoglobin
ICOEPH: International Center for Occupational, Environmental and Public Health
OP: Organophosphate
PchE: Plasma Cholinesterase
PL: Placebo
WHO: World Health Organization
