# Supplementary material for: The use of self-reported symptoms as a proxy for acute organophosphate poisoning after exposure to chlorpyrifos 50% plus cypermethrin 5% among Nepali farmers: a randomized, double-blind, placebo-controlled, crossover study
Source: Environ Health. 2016 Dec 13;15:122. doi: 10.1186/s12940-016-0205-1 (PMC5154153; doi:10.1186/s12940-016-0205-1)
Supplement: Additional file 1: — Number of symptoms and PchE activity at baseline and follow up, and change in symptom sum and percentage change in PchE activity from baseline to follow up for each farmer for the organophosphate spray session (OP) and the placebo spray session (PL). Description: data on symptoms and PchE activity on which conclusions of the manuscript rely. (DOCX 114 kb) [file 12940_2016_205_MOESM1_ESM.docx]

**Additional file 1** Number of symptoms and PchE activity at baseline and follow up, and change in symptom sum and percentage change in PchE activity from baseline to follow up for each farmer for the organophosphate spray session (OP) and the placebo spray session (PL).

| **Farmer** | **OP** |  |  |  |  |  | **PL** |  |  |  |  |  |
| --- | --- | --- | --- | --- | --- | --- | --- | --- | --- | --- | --- | --- |
|  | **Number of symptoms  baseline** | **Number of symptoms follow up** | **Change in symptom  sum** | **PchE baseline (U/mL)** | **PchE follow up (U/mL)** | **% Change in  PchE activity (U/mL)** | **Number of symptoms  baseline** | **Number of symptoms follow up** | **Change in symptom sum** | **PchE baseline (U/mL)** | **PchE follow up (U/mL)** | **% Change in PchE activity (U/mL)** |
| 1 | 1 | 1 | 0 | 1.56 | 1.57 | 0.64 | 2 | 2 | 0 | 1.45 | 1.61 | 11.03 |
| 2 | 6 | 7 | 1 | 2.25 | 2.21 | -1.78 | 0 | 5 | 5 | 2.43 | 2.37 | -2.47 |
| 3 | 0 | 0 | 0 | 1.76 | 1.86 | 5.68 | 0 | 0 | 0 | 2.09 | 1.90 | -9.09 |
| 4 | 2 | 2 | 0 | 1.37 | 1.45 | 5.84 | 0 | 2 | 2 | 1.19 | 1.23 | 3.36 |
| 5 | 1 | 1 | 0 | 1.58 | 1.44 | -8.86 | 0 | 0 | 0 | 1.49 | 1.38 | -7.38 |
| 6 | 1 | 0 | -1 | 1.78 | 1.72 | -3.37 | 0 | 0 | 0 | 1.65 | 1.61 | -2.42 |
| 7 | 0 | 1 | 1 | 1.68 | 1.62 | -3.57 | 4 | 1 | -3 | 1.47 | 1.36 | -7.48 |
| 8 | 0 | 0 | 0 | 1.88 | 1.87 | -0.53 | 0 | 0 | 0 | 2.06 | 2.01 | -2.43 |
| 9 | 0 | 0 | 0 | 1.29 | 1.15 | -10.85 | 0 | 3 | 3 | 1.37 | 1.39 | 1.46 |
| 10 | 0 | 0 | 0 | 2.22 | 2.23 | 0.45 | 2 | 1 | -1 | 2.24 | 2.42 | 8.04 |
| 11 | 0 | 0 | 0 | 1.71 | 1.65 | -3.51 | 0 | 0 | 0 | 1.48 | 1.48 | 0.00 |
| 12 | 0 | 1 | 1 | 1.52 | 1.58 | 3.95 | 0 | 0 | 0 | 1.41 | 1.44 | 2.13 |
| 13 | 1 | 0 | -1 | 1.37 | 1.59 | 16.06 | 0 | 0 | 0 | 1.45 | 1.57 | 8.28 |
| 14 | 1 | 2 | 1 | 1.09 | 1.08 | -0.92 | 0 | 1 | 1 | 1.00 | 1.04 | 4.00 |
| 15 | 0 | 0 | 0 | 1.05 | 1.14 | 8.57 | 0 | 1 | 1 | 0.98 | 0.91 | -7.14 |
| 16 | 7 | 2 | -5 | 1.34 | 1.35 | 0.75 | 2 | 3 | 1 | 1.42 | 1.37 | -3.52 |
| 17 | 2 | 1 | -1 | 0.88 | 0.90 | 2.27 | 5 | 1 | -4 | 0.95 | 1.01 | 6.32 |
| 18 | 5 | 0 | -5 | 1.78 | 1.91 | 7.30 | 0 | 1 | 1 | 1.70 | 1.65 | -2.94 |
| 19 | 0 | 0 | 0 | 1.58 | 1.75 | 10.76 | 0 | 0 | 0 | 1.80 | 1.79 | -0.56 |
| 20 | 0 | 0 | 0 | 1.28 | 1.27 | -0.78 | 0 | 0 | 0 | 1.24 | 1.46 | 17.74 |
| 21 | 0 | 0 | 0 | 1.34 | 1.47 | 9.70 | 0 | 0 | 0 | 1.28 | 1.34 | 4.69 |
| 22 | 2 | 1 | -1 | 1.44 | 1.37 | -4.86 | 1 | 0 | -1 | 1.36 | 1.45 | 6.62 |
| 23 | 0 | 1 | 1 | 1.11 | 1.14 | 2.70 | 0 | 1 | 1 | 1.27 | 1.28 | 0.79 |
| 24 | 3 | 1 | -2 | 1.33 | 1.36 | 2.26 | 0 | 0 | 0 | 1.39 | 1.37 | -1.44 |
| 25 | 0 | 0 | 0 | 1.40 | 1.42 | 1.43 | 0 | 3 | 3 | 1.53 | 1.61 | 5.23 |
| 26 | 0 | 0 | 0 | 2.49 | 2.53 | 1.61 | 1 | 2 | 1 | 1.98 | 2.14 | 8.08 |
| 27 | 2 | 3 | 1 | 1.20 | 1.12 | -6.67 | 0 | 2 | 2 | 1.42 | 1.43 | 0.70 |
| 28 | 1 | 1 | 0 | 0.96 | 1.07 | 11.46 | 2 | 3 | 1 | 0.88 | 0.97 | 10.23 |
| 29 | 0 | 0 | 0 | 1.48 | 1.49 | 0.68 | 0 | 0 | 0 | 1.36 | 1.45 | 6.62 |
| 30 | 0 | 0 | 0 | 1.65 | 1.66 | 0.61 | 1 | 1 | 0 | 1.55 | 1.60 | 3.23 |
| 31 | 1 | 4 | 3 | 2.35 | 2.19 | -6.81 | 4 | 1 | -3 | 2.39 | 2.44 | 2.09 |
| 32 | 1 | 3 | 2 | 1.33 | 1.40 | 5.26 | 1 | 2 | 1 | 1.29 | 1.34 | 3.88 |
| 33 | 2 | 5 | 3 | 1.54 | 1.62 | 5.19 | 1 | 3 | 2 | 1.59 | 1.59 | 0.00 |
| 34 | 0 | 0 | 0 | 1.45 | 1.56 | 7.59 | 1 | 0 | -1 | 1.47 | 1.60 | 8.84 |
| 35 | 1 | 1 | 0 | 1.50 | 1.55 | 3.33 | 0 | 1 | 1 | 1.64 | 1.69 | 3.05 |
| 36 | 0 | 0 | 0 | 2.70 | 2.62 | -2.96 | 0 | 0 | 0 | 2.64 | 2.68 | 1.52 |
| 37 | 1 | 0 | -1 | 1.64 | 1.67 | 1.83 | 2 | 0 | -2 | 1.66 | 1.52 | -8.43 |
| 38 | 0 | 0 | 0 | 1.86 | 1.78 | -4.30 | 0 | 0 | 0 | 1.84 | 1.77 | -3.80 |
| 39 | 0 | 0 | 0 | 1.30 | 1.37 | 5.38 | 0 | 0 | 0 | 1.62 | 1.69 | 4.32 |
| 40 | 0 | 0 | 0 | 2.33 | 2.43 | 4.29 | 0 | 0 | 0 | 2.45 | 2.49 | 1.63 |
| 41 | 1 | 3 | 2 | 1.32 | 1.33 | 0.76 | 2 | 0 | -2 | 1.18 | 1.26 | 6.78 |
| 42 | 0 | 0 | 0 | 1.71 | 1.60 | -6.43 | 0 | 0 | 0 | 1.66 | 1.51 | -9.04 |
